# Supplementary material for: Butyrate and Class I Histone Deacetylase Inhibitors Promote Differentiation of Neonatal Porcine Islet Cells into Beta Cells
Source: Cells. 2021 Nov 19;10(11):3249. doi: 10.3390/cells10113249 (PMC8621544; doi:10.3390/cells10113249)
Supplement: Supplementary file 1 [file cells-10-03249-s001.zip › cells-1446859-supplementary.pdf]

Supplements

Supplemental Figure 1

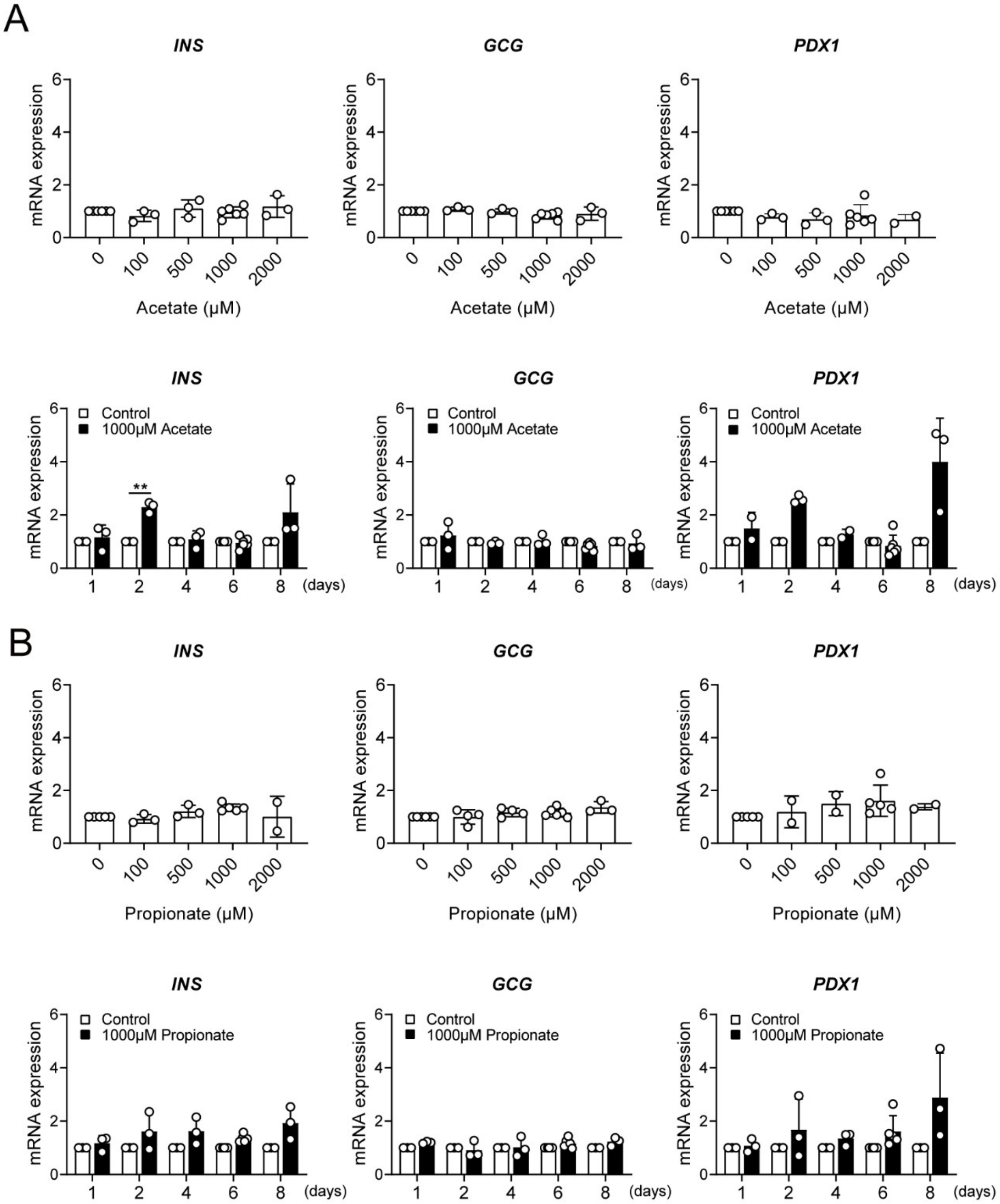

Supplemental Figure S1. Relative gene expression of of insulin (*INS*), glucagon (*GCG*) and *PDX1* detected by RT-qPCR in NPICCs after incubation with acetate (10–2000 µM) or propionate (10–2000 µM) for six days and 1000 µM acetate or 1000 µM propionate for one to eight days. (**A and B**) Time course and dose-dependence of acetate and propionate treatment. Cells without butyrate exposure were used as controls. Data from three to six independent experiments are presented as mean ± SD. ** $p < 0.01$ *vs.* control groups. .

Supplemental Figure 2

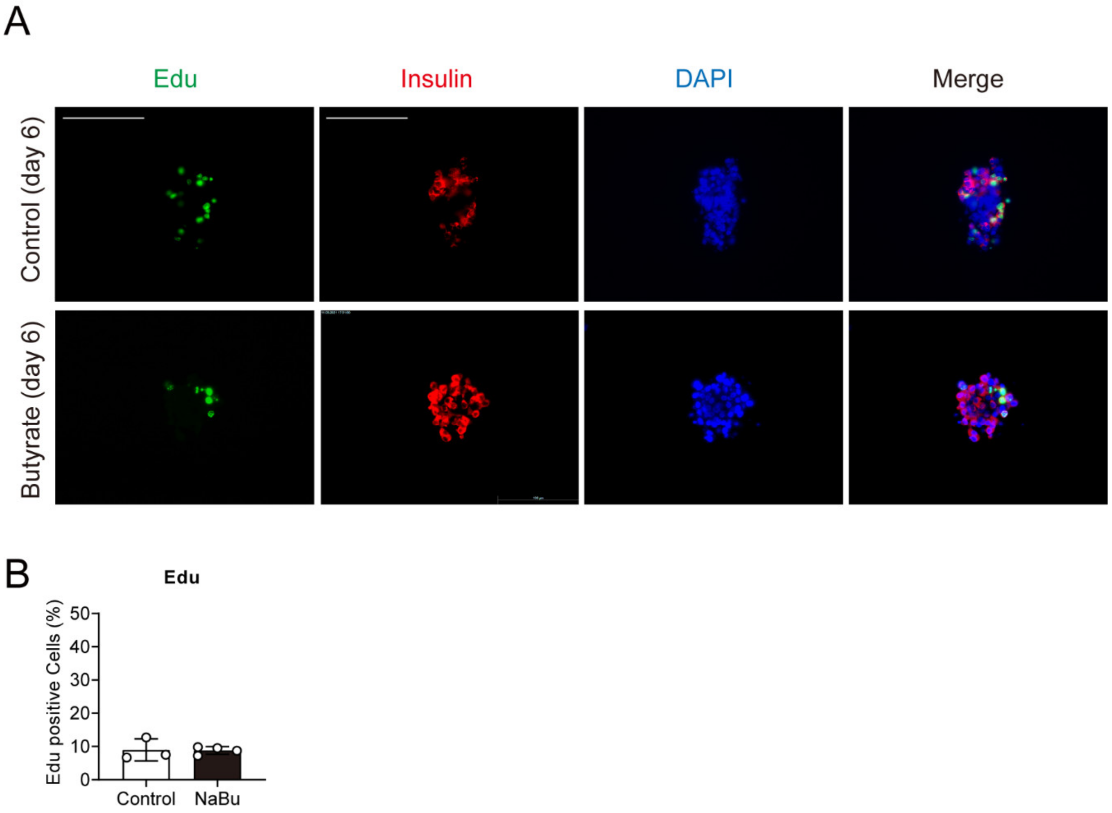

Supplemental Figure S2. The percentage of proliferating cells is not affected by butyrate treatment. NICCs were cultured in the presence of Edu (10 µM). Proliferation was measured by Edu staining (green) and co-staining for insulin (red) and nuclei (blue). (**A**) Representative images are shown. (**B**) Percentage of Edu-positive cells in relation to the total cell count. Scale bars = 100 µm.
